# The antimicrobial volatile power of the rhizospheric isolate *Pseudomonas donghuensis* P482

**DOI:** 10.1371/journal.pone.0174362

**Published:** 2017-03-30

**Authors:** Adam Ossowicki, Sylwia Jafra, Paolina Garbeva

**Affiliations:** 1 Laboratory of Biological Plant Protection, Department of Biotechnology, Intercollegiate Faculty of Biotechnology of University of Gdansk and Medical University of Gdansk, Gdansk, Poland; 2 Department of Microbial Ecology, Netherlands Institute of Ecology (NIOO-KNAW), Wageningen, The Netherlands; Leibniz-Institute of Vegetable and Ornamental Crops, GERMANY

## Abstract

Soil and rhizosphere bacteria produce an array of secondary metabolites including a wide range of volatile organic compounds (VOCs). These compounds play an important role in the long-distance interactions and communication between (micro)organisms. Furthermore, bacterial VOCs are involved in plant pathogens inhibition and induction of soil fungistasis and suppressivenes. In the present study, we analysed the volatile blend emitted by the rhizospheric isolate *Pseudomonas donghuensis* P482 and evaluated the volatile effect on the plant pathogenic fungi and bacteria as well as one oomycete. Moreover, we investigated the role of the GacS/GacA system on VOCs production in *P*. *donghuensis* P482. The results obtained demonstrated that VOCs emitted by *P*. *donghuensis* P482 have strong antifungal and antioomycete, but not antibacterial activity. The production of certain volatiles such as dimethyl sulfide, S-methyl thioacetate, methyl thiocyanate, dimethyl trisulfide, 1-undecan and HCN is depended on the GacS/GacA two-component regulatory system. Apparently, these compounds play an important role in the pathogens suppression as the gacA mutant entirely lost the ability to inhibit via volatiles the growth of tested plant pathogens.

## Introduction

Volatile organic compounds (VOCs) are commonly produced by all organisms including bacteria, fungi and plants. They are low molecular mass molecules (<300 Da) able to penetrate soil and rhizosphere. VOCs synthesized by soil and plant-associated microorganisms have been shown to suppress the growth of plant pathogenic microorganisms indicating that these compounds could be one of the important mechanisms for biological control of plant diseases [1,2].

Recent studies, demonstrated that VOCs can also mediate a variety of interactions between microorganisms and their environment [3]. Indeed, in nature, volatiles are considered to mediate or participate in different intra- and interspecies communication [4,5]. Microbial volatiles can induce systemic resistance of plants or modulate plant growth [6–8]. In bacterial-bacterial interactions they can affect quorum sensing, biofilm formation, secondary metabolites production, antibiotic resistance and virulence [3,5,9,10].

Many volatiles emitted by soil bacteria have antifungal activities and reduce hyphal extension as well as hyphal biomass [4,9]. Hence, bacterial volatiles are considered as a major contributor to soil fungistasis [11,12].

*Pseudomonas* species, which are frequently isolated from soil and rhizosphere are well known as plant growth-promoting rhizobacteria (PGPR) that positively affect plant growth and health by inhibiting the growth of plant pathogens and/or by inducing systemic resistance (ISR) to plant [13]. These bacteria produced numerous secondary metabolites, which have been studied for their antimicrobial activity towards fungi and oomycetes and, to a lesser extent, towards bacteria [13–18]. Among these antimicrobials, *Pseudomonas* strains produce an array of volatile bioactive metabolites, including hydrogen cyanide (HCN), which may participate in the inhibition of many metalloenzymes [19–22].

The production of secondary metabolites in many bacteria, as well as in *Pseudomonas*, is often regulated by GacS/GacA two-component regulatory system [23]. This system consists of a membrane-bound sensory kinase GacS (which senses yet unknown biotic and abiotic signals) and a transcriptional response regulator GacA [24]. Genetically obtained mutants in the gene encoding one of these proteins result in the loss of the antimicrobials production including HCN [25,26]. However, it was also discovered that in *P*. *fluorescens* SBW25 GacS mutants show enhanced antimicrobial activity [27].

*Pseudomonas donghuensis* P482, used in this study, is a tomato rhizosphere isolate belonging to the recently established new species of *Pseudomonas* genus [28]. *P*. *donghuensis* P482 is capable of inhibiting the growth of several plant pathogens, including the stone fruit pathogen *P*. *syringae* [29] and the various strains of soft rot *Enterobacteriaceae* from the genus of *Dickeya* and *Pectobacterium* (formerly *Erwinia*) [29]. The genetic background of the observed antibacterial activity against *Dickeya* and *Pectobacterium* has been recently unveiled [18]. *P*. *donghuensis* P482 also inhibits the growth of the fungal plant pathogen *Rhizoctonia solani* [30], yet, little is known about antifungal properties of this strain, as well as the inhibitory capacity of the VOCs blend produced by *P*. *donghuensis* P482 on the fungal and bacterial pathogens.

In this study, the VOCs blend emitted by *P*. *donghuensis* P482 strain was analysed and evaluated for its effect on the plant pathogenic fungi and oomycete as well as on phylogenetically different bacteria. Furthermore, we investigated the role of the GacS/GacA system in regulation of *P*. *donghuensis* P482 VOCs production.

The results obtained demonstrated that VOCs emitted by *P*. *donghuensis* P482 have strong antifungal and antioomycete, but not antibacterial activity. Furthermore, our results revealed that the production of certain antifungal volatiles is depended the GacS/GacA two-component regulatory system.

## Material and methods

### Strains and growth conditions

Bacterial strains used in this study are listed in Table 1. All strains were maintained on 1/10 TSB agar plates (5.0 g L^-1^ NaCl, 1.0 g L^-1^ KH2PO4; 3 g L^-1^ oxoid tryptic soy broth (TSBA); 20 g L^-1^ Merck Agar, pH 6.5; Garbeva and de Boer, 2009) or LB (Novagen, Germany) at 25°C, when necessary supplemented with kanamycin (30 μg ml^-1^ Sigma-Aldrich, USA). For the growth of the mating strain *E*. *coli* ST18 the medium was supplemented with 5-aminolevulonic acid (ALA) 30 μg ml^-1^ (Sigma-Aldrich, USA) and the bacteria were grown at 37°C. All fungi and oomycetes cultures were pre-grown on 1/2 potato dextrose agar (PDA; 29 g L^−1^ Oxoid CM139; [31]) for one-week prior to use them in the experiments.

**Table 1:** The list of the bacterial, fungal and oomycetes strains used in this study.

Strain	Origin/ features	reference
*Pseudomonas donghuensis* P482	Tomato rhizosphere isolate—a wild type strain	[29]
KN3318	*P*. *donghuensis* P482 mutant carrying an inbuilt pKNOCK-Km suicide vector in *gacA* gene (locus BV82_3318); Km^R^ BV82_3318::pKnock-Km	This study
*Escherichia coli* ST18	Donor strain for diparental mating; *pro thi hsdR*^+^ Tp^R^ Sm^R^; Chromosome::RP4-2 Tc::Mu-Kan::Tn7/λpir *ΔhemA*	[32]
*Escherichia coli* DH5α	Cloning strain; *fhuA2 lac(del)U169 phoA glnV44 Φ80' lacZ(del)M15 gyrA96 recA1 relA1 endA1 thi-1 hsdR17*,high efficiency transformation strain	[33]
*Agrobacterium* sp. AD140	NIOO-KNAW collection	[9]
*Pseudomonas fluorescens* AD21	NIOO-KNAW collection	[9]
*Rhizoctionia solani* AG2.2IIIB	Plant pathogen NIOO-KNAW collection	[9]
*Fusarium culmorum PV*	Plant pathogen NIOO-KNAW collection	[9]
*Verticulum dahliae* JR	Plant pathogen NIOO-KNAW collection	[9]
*Pythium ultimum* P17	Plant pathogen NIOO-KNAW collection	[9]

### Construction of the *gacA* mutant

A locus of *gacA* gene was identified and annotated in *P*. *donghuensis* P482 genome (JHTS00000000.1; [30]) based on ten sequences of the homologous genes present in pseudomonas.com database [34]. The mutation was introduced using pKnock-Km system [35] as previously described by Krzyzanowska et al. [18]. Briefly, the fragment of interest (413 bp of *gacA* gene) was amplified with Hot Start II Phusion DNA polymerase (Thermo Scientific, USA) and the primer pair gaca_insF/gaca_insR (S1 Fig) in conditions recommended by the supplier. A PCR product obtained was cloned between the KpnI/SalI restriction sites of the pKNOCK-Km suicide vector [35]. The resulting construct pKN3318 was introduced into *E*. *coli* ST18 [31] via electroporation (Gene Pulser Xcell^™^, Bio-Rad). Subsequently the positive *E*. *coli* transformants were selected on LB medium with ALA and kanamycin. The generated plasmid was transferred to *P*. *donghuensis* P482 by biparental mating. Donor carrying pKN3318 plasmid and recipient strains were grown overnight. From each culture 2 ml was spin down and rinsed twice with 0,9% NaCl (Avantor, Poland). Pellets were suspended in 25 μl LB, pooled together and transferred as a droplet onto LB agar plate with ALA then incubated 20h at 37°C. The macro-colony established on the plate was collected and re-suspended in 1 ml LB medium. One hundred microliter aliquots of the suspension and serial dilutions (10^−1^, 10^−2^, 10^−3^) were plated on LB agar supplemented with 30μg·ml^−1^ kanamycin but lacking ALA, thus preventing the growth of the *E*.*coli* ST18. Nine *P*. *donghuensis* P482 transconjugants obtained were screened for the presence of the pKNOCK-Km insertion with the primer pair gaca_outF/gaca_outR (S1 Table). To confirm that the suicide vector had incorporated into the target loci, genomic DNA of each mutant was used as template in a sequencing reaction with primer F_outof_pKNOCK. The results obtained enabled mapping of the pKNOCK-Km insertion site to the genome of the *P*. *donghuensis* P482 strain. Two of obtained mutants (KN3318-1 and KN3318-2) were sequenced by Oligo.pl (Warsaw, Poland) that finally confirmed presence of pKnock-Km insert in *gacA* gene (locus BV82_3318). One of the sequenced strains (KN3318-1) was chosen and used for further experiments as KN3318.

### Effect of volatiles produced by *P*. *donghuensis* P482 on fungi and oomycete growth

To investigate the VOCs effect on the growth of the tested fungi and oomycete bottom-top approach was applied as described previously by Garbeva et al., [9]. Bacteria suspensions washed with 10 mM phosphate buffer were spread at the bottom part of the plates on 20 ml 1/10 TSB agar. For a control, a sterile phosphate buffer was plated. The top part (the lid) of Petri dish contained 12 ml water agar (WA) and the 6 mm-in-diameter PDA disc with fungi/oomycete was placed in the center; the plates were sealed with the parafilm, and incubated at 25°C.

When the effect of the pure compounds: S-methyl thioacetate (MTA), dimethyl disulfide (DMDS) or dimethyl trisulfide (DMTS) (Sigma-Aldrich, Germany), was tested, the bottom part of the plate contained a paper disc soaked with one or two (mixed) pure compounds. In every treatment with single compound 1 μl was added (MTA- 11.4 μM, DMDS– 11.1 μM, DMTS– 9.5 μM), for the mix 1 μl of each compound was used. The plates were immediately sealed with the parafilm and incubated at 25°C.

### Effect of volatiles produced by *P*. *donghuensis* P482 on bacteria growth

Two compartment Petri dishes with 10 ml of 1/10 TSB per each part were used to evaluate the effect of VOCs produced by *P*. *donghuensis* P482 on two bacterial species: *Agrobacterium* sp. AD140 and *P*. *fluorescens* AD21. One side of the plate was inoculated with 50 μl of 10^5^ cfu ml^-1^ *P*. *donghuensis* P482 and incubated for 2 days. Afterwards overnight liquid cultures of the model strains AD140 and AD21 were added at the other compartment of the plate at different bacteria densities (10μl drops). Enumeration of AD140 and AD21 was performed under the binocular after overnight incubation in 25°C, all the experimental setups were prepared in triplicates.

### Hydrogen cyanide production

For hydrogen cyanide (HCN) detection in the headspace of *P*. *donghuensis* P482, a method adapted from Castric and Castric [36] was used. Sterile Whatmann paper (3 mm thick and size 128 x 86 mm) was soaked with suspension containing 5 mg ml^-1^ of copper(II) ethyl acetoacetate (Sigma-Aldrich, USA) and 4,4'-methylenebis-(N,N-dimethylaniline) (Sigma-Aldrich, USA) in chloroform and dried in sterile conditions. One hundred μl of 1/10 TSB medium was poured into the wells of the 96-well plate. Three different treatments were prepared i) 1/10 TSB without any supplementation, ii) 1/10 TSB supplemented with 50μM FeCl_3_ (Avantor, Poland), and iii) 1/10 TSB supplemented with 20 μM glycine (Sigma-Aldrich, USA). All the treatments were prepared in duplicates. Wells were inoculated with 1 μl overnight culture of *P*. *donghuensis* P482, KN3318 or the HCN non-producer *E*. *coli* DH5α. All bacterial cultures were diluted to 10^8^ cfu ml^-1^. Plate was covered with freshly prepared and dried Whatmann paper and the plastic cover. The plate was pressed using six large binder clips and incubated 24 h at 25°C. After this time the development of the blue color, indicating HCN production was examined.

### Volatile trapping and GC-MS analysis

*P*. *donghuensis* P482 and KN3318 mutant were grown in triplicates on 1/10 TSB agar medium, non-inoculated medium was used as a control. Emitted volatile compounds were collected in the steel traps with 150 mg Tenax TA and 150 mg Carbopack B (Markes International Ltd, Llantrisant, UK) applied to the specially adapted glass petri dishes [9]. Traps were removed, capped and stored at 4°C until GC-Q-TOF analysis. Volatiles were desorbed from the traps using an automated thermodesorption unit (model UnityTD-100, Markes International Ltd., Llantrisant, UK) at 210°C for 12 min (Helium flow 50 mL/min) and trapped on cold trap at -10°C. The trapped volatiles were introduced into the GC-QTOF (model Agilent 7890B GC and the Agilent 7200A QTOF, Santa Clara, USA) by heating the cold trap for 3 min to 280°C with split ratio set to 1:20. The column used was a 30 × 0.25 mm ID RXI-5MS, film thickness 0.25 μm (Restek 13424–6850, Bellefonte, PA, USA). Temperature program used was as follows: 39°C for 2 min, from 39°C to 95°C at 3.5°C/min, then to 165°C at 6°C/min, to 250°C at 15°C/min and finally to 300°C at 40°C/min, hold 20 min. The volatiles were detected by the MS operating at 70 eV in EI mode. Mass spectra were acquired in full scan mode (30–400 amu, 4 scans/s). Identification of metabolites was performed using NIST-MS Search and accurate mass and spectra match factor using NIST 2014 V2.20 (National Institute of Standards and Technology, USA, http://www.nist.gov) and Wiley 9th edition spectral libraries and by their linear retention indexes (lri). The lri values were compared with those found in the NIST and in the in-house NIOO lri database. The LRI values were calculated using an alkane calibration mix before the measurements in combination with AMDIS 2.72 (National Institute of Standards and Technology, USA).

### Statistical analysis

For the metabolomics analysis the acquired raw MS data was extracted to m/z format with MassHunter Qualitative Analysis Software V B.07.00 (Agilent Technologies, Santa Clara, CA, USA) and processed with MZMine V 2.21 (Copyright © 2005–2012 MZmine Development Team, [37]) to create m/z and peak intensity table that could be used as input file to MetaboAnalyst 3.0 software (http://www.metaboanalyst.ca/MetaboAnalyst [38]). Before the statistical analysis, data was filtered using Interquantile range (IQR) and normalized by the log transformation with automatic scaling. On the pretreated dataset following analyses were performed: clustering using partial least squares—discriminant analysis (PLS-DA), One-way Analysis of Variance (ANOVA) and Hierarchical Clustering. The statistical analyses of fungal antagonistic tests and bacterial enumeration were carried out with Excel using ANOVA and Student’s t-Test. Data were considered to be statistically different at p≤ 0.05.

## Results

### Effect of volatiles emitted by *P*. *donghuensis* P482 on the fungal, oomycete and bacterial growth

**Fig 1:**
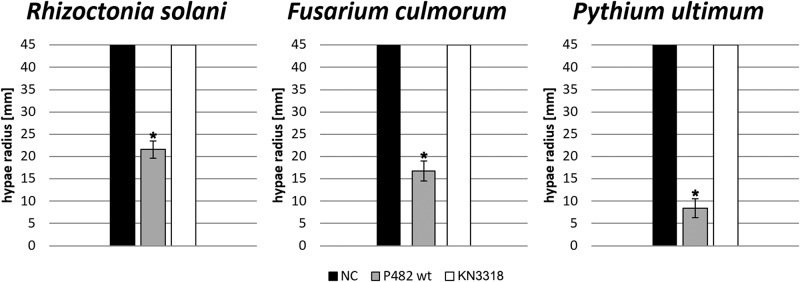
Average hyphae radius after 4 days (*Rhizoctonia solani*), 7 days (*Fusarium cilmorum*) and 5 days (*Pythium ultimum*) of incubation under influence of volatiles emitted by *P*. *donghuensis* P482 wt, KN3318 mutant and non-treated control (NC). Significant difference between sample and control are indicated by asterisk (one-way ANOVA), error bars represents standard deviation of the mean.

The growth of the 2 fungal (*R*. *solani* AG2.2IIIB and *F*. *culmorum* PV) and one oomcycete (*P*. *ultimum* P17) pathogens was significantly inhibited when exposed to the volatiles emitted by *P*. *donghuensis* P482 wt (Fig 1). The hyphal inhibition varied from about 50 to 80% reduction of fungal and oomycete growth as compared to the control. The strongest inhibition (>80%) was observed on the oomycete pathogen *P*. *ultimum* P17. The lowest, yet still significant (about 50%) growth reduction was observed on the *R*. *solani* strain.

The growth of *V*. *dahliae* in the given experimental condition was very slow and after 14 days of incubation the hypha expanded only about 5–6 mm in case of the non-treated control. However, when exposed to volatiles emitted by *P*. *donghuensis* P482 the growth of *V*. *dahliae* was completely suppressed (S2 Fig).

**Fig 2:**
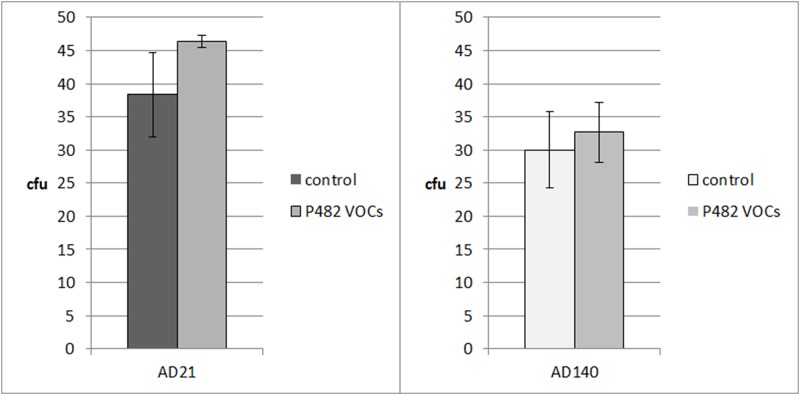
Number of colony forming units (cfu) of *Pseudomonas fluorescens* AD21 and *Agrobacterium* sp. AD140 after overnight exposure to volatiles emitted by *P*. *donghuensis* P482 and control without bacterial volatiles. Error bars represents standard deviation of the mean.

The exposure of bacterial strains *Agrobacterium* sp. AD 140 and *Pseudomonas fluorescens* AD21 to the volatiles emitted by *P*. *donghuensis* P482 did not cause any significant difference in their growth (Fig 2).

### GacA deficiency abolished *P*. *donghuensis* P482 volatiles-mediated inhibition of the tested fungal and oomycete pathogens

The results obtained with the GacA-deficient mutant KN3318 revealed that the mutant lost entirely its volatile-mediated inhibition resulting in hypha extension of the tested fungal and oomycete pathogens similar to that of the controls (Fig 1). In case of the tested strain of *V*. *dahliae* the growth of hypha was as slow as observed for the untreated control (S2 Fig). However, when exposed to volatiles emitted by KN3318, the change in a phenotype of *V*. *dahliae* mycelium was observed in comparison to the non-treated control. The untreated mycelium was black in the center but in the case of exposure to KN3318- emitted volatiles it remains white (S2 Fig). Microscopic observation revealed that this difference in coloration is due to the formation of microsclerotia by the fungus (pigmented aggregates). This phenomenon was not observed under the exposure to *P*. *donghuensis* P482 wt.

### GC-MS and metabolomics analysis of volatiles emitted by *P*. *donghuensis* P482

**Fig 3:**
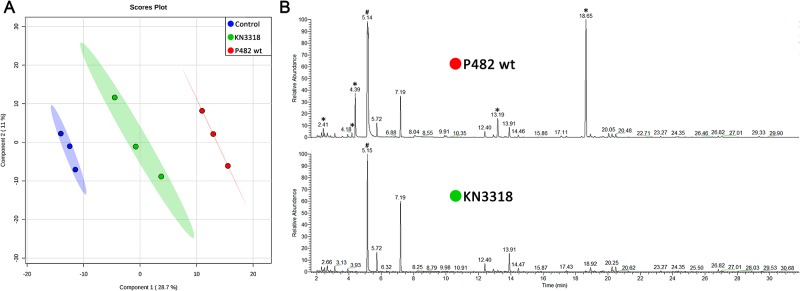
Data obtained from MetaboAnalyst 3.0 and GC-MS results. (A) PLS-DA plot of VOC’s produced by *P*. *donghuensis* P482 and KN3318 mutant with media as a control (triplicates); (B) TIC chromatographs from GC-MS analysis showing different peaks between *P*. *donghuensis* P482 and KN3318 mutant. Significant peaks that differ between wild type and mutant are indicated by asterisk. Dimethyl disulfide (DMDS) is indicated by “#”.

The volatolomic analysis of *P*. *donghuensis* P482 wt and its GacA-deficient mutant KN33318 revealed that their volatile profiles differ. Clear separations between control, wild type and mutant were obtained in PSL-DA score plot (Fig 3A). Five major peaks with RT 2.41, 4.18, 4.39, 13.19 and 18.65 were not detected in the headspace of KN3318 mutant and the control consisting only of media without bacteria (Fig 3B and 3C). The volatiles emitted by *P*. *donghuensis* P482 wt strain but not present in the KN3318 mutant headspace were identified as dimethyl sulfide, S-methyl thioacetate, methyl thiocyanate, dimethyl trisulfide and 1-undecan (Table 2). Two compounds namely dimethyl trisulfide and S-methyl thioacetate were confirmed with commercially available authentic standards. In both *P*. *donghuensis* P482 wt and the GacA KN33318 mutant the highly abundant peak with RT 5.14 was identified as dimethyl disulfide.

**Table 2:** Volatile compounds identified in the headspace *P*. *donghuensis* P482 wt not present in the KN3318 gacA mutant culture.

RT	Compound name	ERI	Formula
2.41	dimethyl sulfide	540	C_2_H_6_S
4.18	S-methyl thioacetate	701	C_3_H_6_OS
4.39	methyl thiocyanate	709	C2H3NS
13.19	dimethyl trisulfide	966	C_2_H_3_S_3_
18.65	1-undecan	1091	C_11_H_22_
N/A*	hydrogen cyanide	*N/A*	HCN

RT- retention time in min and ERI- experimental retention index;

* Hydrogen cyanide could not be detected/measured using GC-QTOF and was measured as described by Castric and Castric [36]

### Effect of S-methyl thioacetate, dimethyl disulfide and dimethyl trisulfide on fungal and oomycete growth

**Fig 4:**
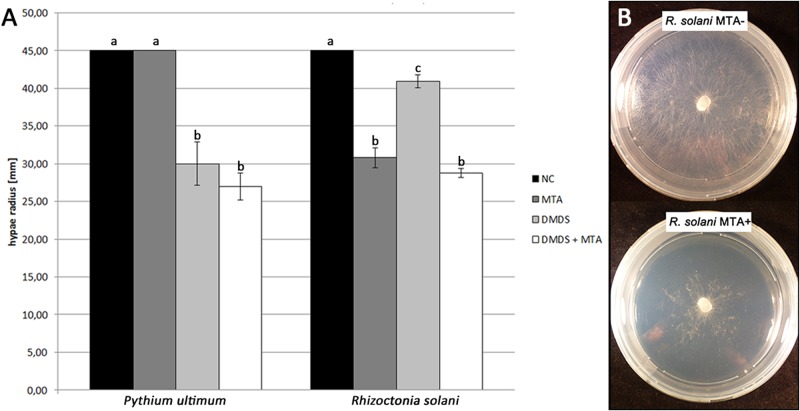
(A) Average hyphae radius of *P*. *ultimum* and *R*. *solani* after 4 days of incubation exposed and not exposed (NC) to pure volatile organic compounds: S-methyl thioacetate (MTA), dimethyl disulfide (DMDS) and combination of MTA and DMDS. Significant difference between samples are indicated by different letters (one-way ANOVA, post hoc t-test p < 0.05), error bars represents standard deviation of the mean. (B) Photos of *R*. *solani* cultures exposed (+) and not exposed (-) to MTA.

Pure S-methyl thioacetate (MTA), found to be produced by *P*. *donghuensis* P482 wt but not by the GacA-deficient KN33318 mutant, was tested on two selected plant pathogens: *R*. *solani* and *P*. *ultimum* using the bottom-top approach. For oomycete *P*. *ultimum* there was no significant difference between pathogen treated with MTA and non-treated control but in case of *R*. *solani* the growth inhibition was significant and comparable to the effect of volatile blend produced by *P*. *donghuensis* P482 wt (Fig 4) indicating that this particular compound is important for the growth inhibition of *R*. *solani*. The pure compound dimethyl disulfide caused significant inhibition on both *R*. *solani* and *P*. *ultimum*. Slightly stronger inhibition (but not significant) was observed when MTA was applied in combination with dimethyl disulfide (Fig 4). Complete growth inhibition of both *R*. *solani* and *P*. *ultimum* was observed when dimethyl trisulfide was applied (data not shown).

### Hydrogen cyanide production

Production of the hydrogen cyanide (HCN) was detected in all media variants only in *Pseudomonas donghuensis* P482 but not in GacA-deffecient KN3318 mutant or *E*. *coli* (S3 Fig).

Supplementation of the growing medium (1/10 TSB) with 50μM glycine resulted in the darker blue color in comparison to *P*. *donghuensis P482* wt grown only in basal medium or medium supplemented with iron.

## Discussion

Soil and rhizosphere bacteria produce a large amount of secondary metabolites, which have many different physiochemical and biological properties. Beside well documented, soluble antimicrobial compounds bacteria emit a wide range of VOCs that hold a strong inhibitory capacity [5,9,20,39]. In this study, we attempted to determine the VOCs emitted by the rhizospheric isolate *P*. *donghuensis* P482, their antimicrobial activity and the role of the two-component GacS/GacA regulatory system in VOCs production.

Our results revealed that *P*. *donghuensis* P482 strain is emitting VOCs with strong antifungal and antioomycete properties and significantly inhibited the plant pathogens *R*. *solani*, *F*. *culmorum*, *V*. *dahliae* and *P*. *ultimum*. Interestingly GacA-deficient mutant lost entirely the ability to inhibit *via* volatiles the tested plant pathogens. The metabolomics analysis revealed clear differences in volatile profiles between the *P*. *donghuensis* P482 and the GacA mutant KN3318. Five compounds detected only in the headspace of *P*. *donghuensis* P482 but not in the headspace of GacA-deficient mutant were identified as dimethyl sulfide, S-methyl thioacetate, methyl thiocyanate, dimethyl trisulfide and 1-undecan.

The 1-undecan together with dimethyl disulfide were the most abundant volatiles detected in the headspace of *P*. *donghuensis* P482 which is in line with other studies on VOCs produced by plant-associated *Pseudomonas* [20,21]. In the recent study of Hunziker et al [21] the 1-undecene, emitted by plant-associated *Pseudomonas*, was indicated as the main and active compound with antioomycete properties and with strong inhibitory power to hinder *Phytophtora infestans* growth and development [21].

Interestingly, our results revealed that the production of dimethyl disulfide in contrast to the other sulfur containing volatiles namely dimethyl sulfide, S-methyl thioacetate, methyl thiocyanate, and dimethyl trisulfide, was not regulated by the two-component GacS/GacA system, as it was detected both in the headspace of *P*. *donghuensis* P482 wt and GacA deficient mutant (Fig 3B).

The sulfur containing volatile S-methyl thioacetate was tested individually or in combination with dimethyl disulfide for the ability to inhibit the growth of the model plant pathogens *R*. *solani* and *P*. *ultimum*. Individually applied, the S-methyl thioacetate revealed only anti-fungal activity but did not inhibit the growth of the oomycete *P*. *ultimum*. This apparent difference in sensitivity of the oomycete to volatiles may be related to the cell wall composition and structure, which is different from that of fungi.

Pinpointing of the observed effects to a single volatile compound can be complicated, because fungi and oomycetes might react to a blend of volatiles, rather than to single compounds. For example, when S-methyl thioacetate was applied in combination with other sulfur containing volatiles such as dimethyl disulfide or dimethyl triulfide stronger inhibition of both *R*. *solani* and *P*. *ultimum* was observed. Although numerous studies including this one are trying to pinpoint a single volatile compound responsible for antifungal and antioomycete properties most probably the synergistic or additive effect of several compounds are responsible for the strong antimicrobial volatile activity [12,21].

Beside volatile organic compounds many bacteria are producing large amount of inorganic compounds such as hydrogen cyanide or ammonia [8,21,40] already demonstrated to possess strong antimicrobial properties [21,41,42]. Our results revealed that the production of the volatile hydrogen cyanide in *P*. *donghuensis* P482 is also regulated by the two-component GacS/GacA system as reported for other *Pseudomonas* and *Chromobacterium* species [43,44].

While many study tested the effect of bacterial VOCs on various fungi and oomycete relatively few studies have reported on VOCs with antibacterial activities [5,45]. We revealed that the VOCs produced by *P*. *donghuensis* P482 did not influence the growth of the tested rhizosphere isolates *P*. *fluorescens* AD21 and *Agrobacterium* sp. AD140 (nor *Dickeya solani* IFB 102 strain (S. Jafra personal communication)). This is in line with the recent study of Garbeva et al., [46] where *P*. *fluorescens* Pf0-1 was exposed to VOCs produced by 4 phylogenetically different bacterial isolates (*Collimonas pratensis*, *Serratia plymuthica*, *Paenibacillus* sp., and *Pedobacter* sp.) growing in sand containing artificial root exudates. Their results revealed that the bacterial VOCs rather stimulated than inhibited the growth of *P*. *fluorescens* Pf0-1. However, bacterial growth suppression was reported for dimethyl trisulfide emitted by *Pseudomonas* strains against the *Agrobacterium* sp. causing crown-gall diseases [19,47]. Recently, Tyc et al. [45] tested the two commonly produced bacterial VOCs namely dimethyl di- and trisulfide for their effect on bacteria. The experiments revealed strong inhibition on the tested bacterial model organisms, only when applied in the highest tested concentration of 50 μM [45]. However, it is unclear at what concentration these VOCs are produced in the natural environment.

It has been speculated that variations in sensitivity of bacteria to volatiles may possibly be mediated by an ATP-dependent efflux mechanism, which has been investigated for several terpene compounds against *Pseudomonas aeruginosa* [48] as well as the ability of the VOCs to disintegrate the outer membrane [49]. Hence, the outcome of bacterial volatile-mediated interactions can strongly vary depending on the interacting partners.

In conclusion, our work revealed that the rhizospheric isolate *P*. *donghuensis* P482 emits VOCs with strong antifungal and antioomycete activity and it is depended on the GacS/GacA two-component regulatory system. Further studies are needed to explore the antimicrobial volatile power of this isolate under natural conditions e.g. in greenhouse and field experiments.

## Supporting information

S1 Fig Region of pKNOCK-Km insertion in *Pseudomonas donghuensis* P482 genome. Locus BV82_3318 was identified as *gacA* gene. Place of the homologous recombination is indicated by the “X” sign between the plasmid and the sequence, black arrows shows the places of primers hybridization (primers sequences are listed in S1 Table).
(DOCX)

S2 Fig Photography of the V. *dahliae* cultures exposed to the *Pseudomonas* P482 wt volatiles, to the KN3318 mutant volatiles and non-treated control. (DOCX)

S3 Fig Results of HCN detection assay. Change of color from white to blue indicates the production of the hydrogen cyanide.
(DOCX)

S1 Table Oligonucleotides and plasmids used in study, restriction sites are shown in bold. (DOCX)
