# Supplementary material for: The antimicrobial volatile power of the rhizospheric isolate Pseudomonas donghuensis P482
Source: PLoS One. 2017 Mar 30;12(3):e0174362. doi: 10.1371/journal.pone.0174362 (PMC5373542; doi:10.1371/journal.pone.0174362)
Supplement: S1 Fig — Locus BV82_3318 was identified as gacA gene. Place of the homologous recombination is indicated by the “X” sign between the plasmid and the sequence, black arrows shows the places of primers hybridization (primers sequences are listed in S1 Table). (DOCX) [file pone.0174362.s001.docx]

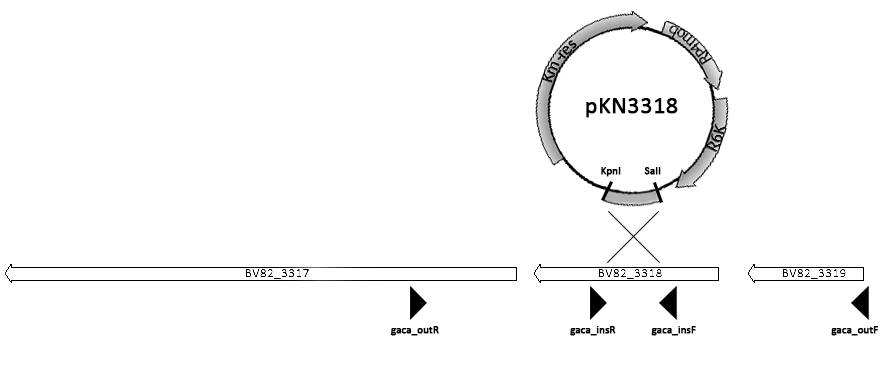


**S1 Fig.** Region of pKNOCK-Km insertion in *Pseudomonas donghuensis* P482 genome. Locus BV82_3318 was identified as *gacA* gene. Place of the homologous recombination is indicated by the “X” sign between the plasmid and the sequence, black arrows shows the places of primers hybridization (primers sequences are listed in table S2).
