# Supplementary material for: The antimicrobial volatile power of the rhizospheric isolate Pseudomonas donghuensis P482
Source: PLoS One. 2017 Mar 30;12(3):e0174362. doi: 10.1371/journal.pone.0174362 (PMC5373542; doi:10.1371/journal.pone.0174362)
Supplement: S2 Fig — (DOCX) [file pone.0174362.s002.docx]

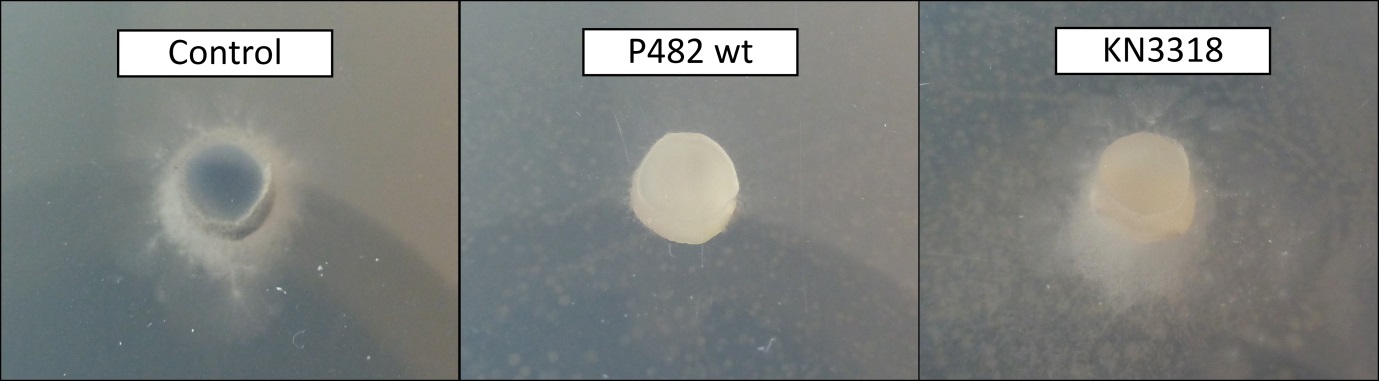


**S2 Fig.** Photography of the V*. dahliae* cultures exposed to the *Pseudomonas* P482 wt volatiles , to the KN3318 mutant volatiles and non-treated control.
