# Supplementary material for: The antimicrobial volatile power of the rhizospheric isolate Pseudomonas donghuensis P482
Source: PLoS One. 2017 Mar 30;12(3):e0174362. doi: 10.1371/journal.pone.0174362 (PMC5373542; doi:10.1371/journal.pone.0174362)
Supplement: S3 Fig — Change of color from white to blue indicates the production of the hydrogen cyanide. (DOCX) [file pone.0174362.s003.docx]

**
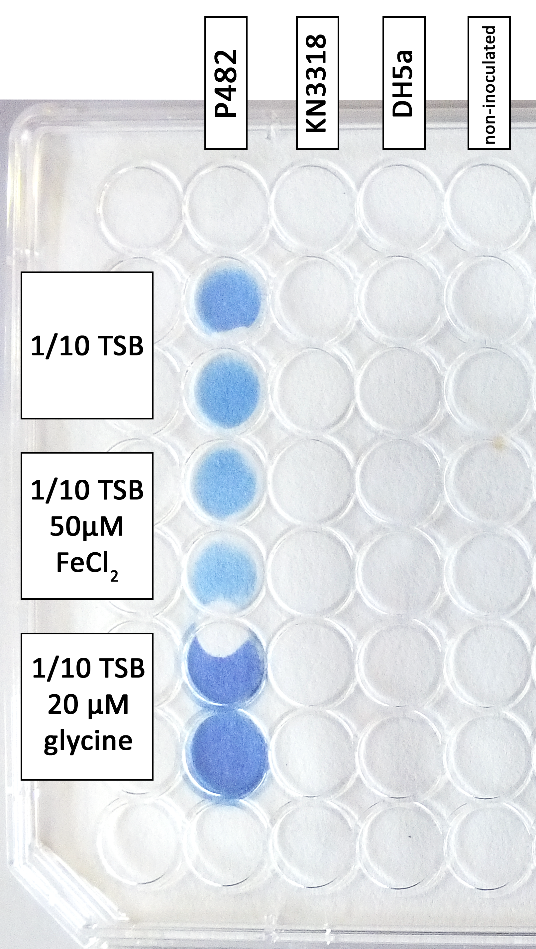
**

**S3 Fig.** Results of HCN detection assay. Change of color from white to blue indicates the production of the hydrogen cyanide.
