# Supplementary material for: The antimicrobial volatile power of the rhizospheric isolate Pseudomonas donghuensis P482
Source: PLoS One. 2017 Mar 30;12(3):e0174362. doi: 10.1371/journal.pone.0174362 (PMC5373542; doi:10.1371/journal.pone.0174362)
Supplement: S1 Table — (DOCX) [file pone.0174362.s004.docx]

S1 Table. Oligonucleotides and plasmids used in study, restriction sites are shown in bold.

| **name** | **Sequence/description** | **restriction site** |
| --- | --- | --- |
| gaca_insF | 5' ATA**GGTACC**ACGTGGTCCTGATGGATGTG 3' | KpnI |
| gaca_insR | 5' ATA**GTCGAC**GATCCGGTAACGGTAAGTATTGAC 3' | SalI |
| gaca_outF | 5' ATA**GGTACC**AAGCAAATGAACGGAATC 3' | KpnI |
| gaca_outR | 5' ATA**GTCGAC**TGCTGTAATAGCTGTCTTCACA 3' | SalI |
| pKNOCK-Km | Suicide plasmid | N/A |
| pKN3318 | pKNOCK-Km with insert | N/A |
